# Dynamic transition of molecular subtypes in relapsed small cell lung cancer treated with multimodal therapy: A case report

**DOI:** 10.1111/1759-7714.14983

**Published:** 2023-05-30

**Authors:** Kengo Yasuda, Tomohiro Haruki, Tatsuya Miyamoto, Yuki Oshima, Shinji Matsui, Yasuaki Kubouchi, Hiroshige Nakamura

**Affiliations:** ^1^ Department of Surgery, Division of General Thoracic Surgery, Faculty of Medicine Tottori University Tottori Japan

**Keywords:** ASCL1, concurrent chemoradiotherapy, NEUROD1, salvage surgery, small cell lung cancer

## Abstract

Several transcription factors in small cell lung cancer (SCLC), including achaete‐scute homolog 1 (ASCL1) and neurogenic differentiation factor 1 (NEUROD1), contribute to rapid tumor growth and early metastatic dissemination. Recent studies suggested that these molecular subtypes represent neuroendocrine differentiation in dynamic SCLC evolution. In the present case, a 62‐year‐old man was diagnosed with limited disease SCLC originating from the right upper lobe. Biopsy specimens were positive for ASCL1 but negative for NEUROD1. Six months after concurrent chemoradiotherapy and prophylactic cranial irradiation, the primary tumor had regrown and salvage surgery was performed. The pathological diagnosis was recurred SCLC, and postoperative histopathology was positive for both ASCL1 and NEUROD1. The patient was subsequently followed up; however, he had multiple bone metastases 9 months after surgery. It was speculated that the shift to NEUROD1‐high expression in tumor cells surviving concurrent chemoradiation therapy may be related to the poor outcome after combined modality treatment.

## Graphical abstract

We report a case of salvage surgery for the local recurrence of small cell lung cancer after concurrent chemoradiation therapy (CCRT). Histological examination of this case confirmed the alterations in molecular subtypes. The shift to NEUROD1‐high expression in tumor cells surviving CCRT may be related to the poor outcome after combined modality treatment.

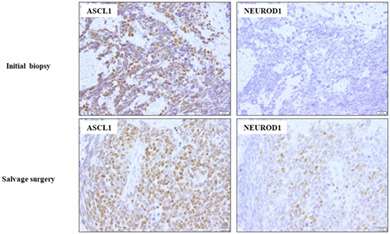

## INTRODUCTION

Small cell lung cancer (SCLC) is a high‐grade neuroendocrine carcinoma that behaves aggressively and has a poor prognosis. Among all SCLC patients, approximately one‐third have limited disease SCLC (LD‐SCLC), and multimodal treatment including chemotherapy and radiotherapy is generally recommended for those patients.^1^, ^2^ Nevertheless, most patients relapse within 2 years of treatment, and approved therapies are effective in less than 20% of patients following relapse.^3^ The natural history of SCLC may include rapid evolution from chemosensitivity to chemoresistance in relapsed cases. Recent studies have suggested that SCLC can be classified into molecular subtypes based on the expression of lineage transcription factors such as achaete‐scute homolog 1 (ASCL1), neurogenic differentiation factor 1 (NEUROD1), POU domain class 2 transcription factor 3 (POU2F3), and transcriptional coactivator YAP1 (YAP1).^4^, ^5^ These transcription factors are reported to play essential roles in the heterogeneity of SCLC cells as well as in different stages of neuroendocrine evolution, including acquired therapeutic resistance.^6^ However, the evidence for neuroendocrine differentiation shifting during SCLC evolution is lacking in the clinical setting.

Here, we report a patient who underwent salvage surgery for relapsed SCLC after concurrent chemoradiotherapy (CCRT). Postoperative histological examination showed that NEUROD1 expression shifted from negative to positive.

## CASE REPORT

**FIGURE 1:**
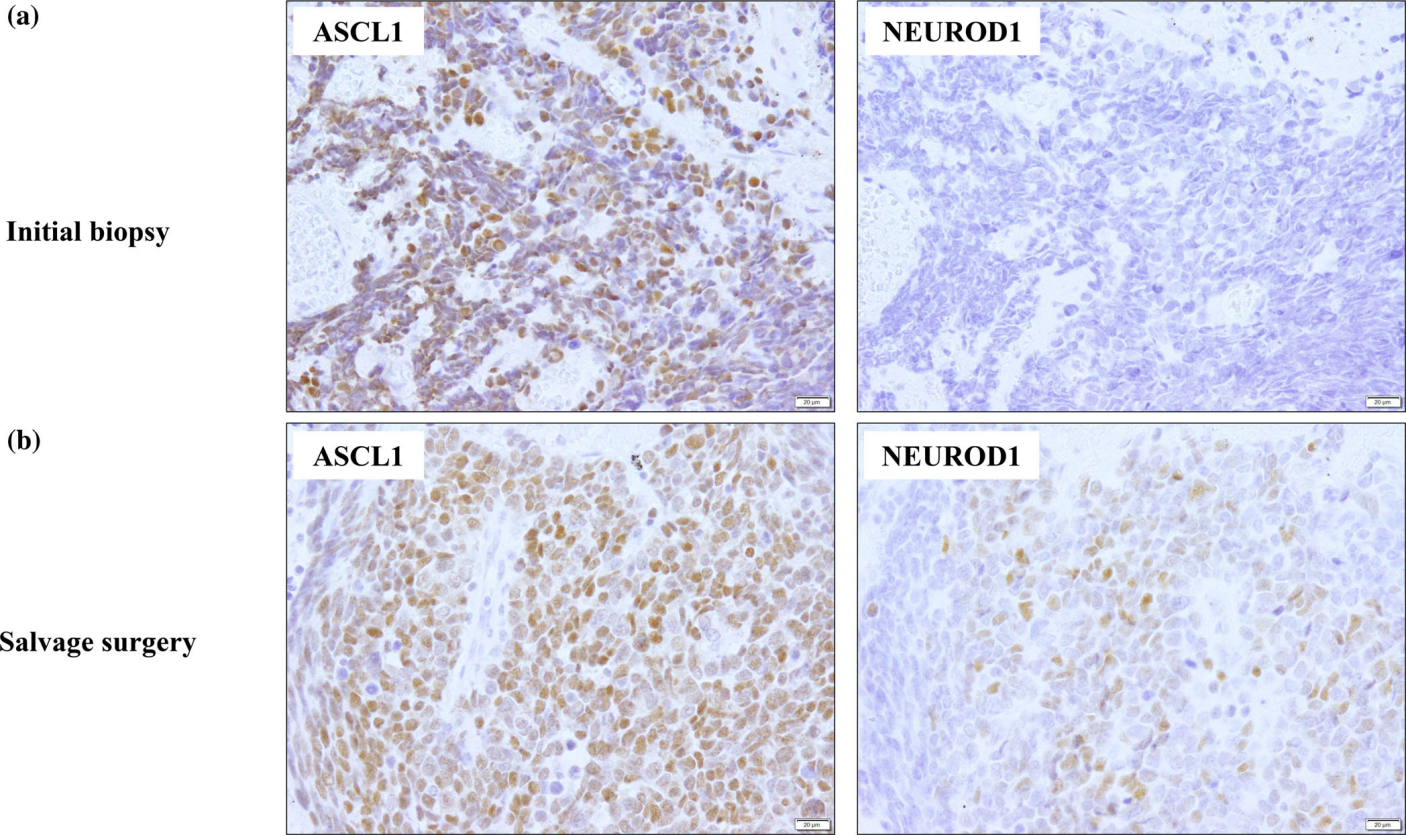
Pathology. (a) The primary tumor was diagnosed as small cell lung cancer (SCLC), being positive for achaete‐scute homolog 1 (ASCL1) and negative for neurogenic differentiation factor 1 (NEUROD1) (magnification, 200×). (b) After salvage surgery, histological examination showed SCLC without mediastinal lymph node metastases. The specimens became positive for ASCL1 and NEUROD1 (magnification, 200×).

A 62‐year‐old man was referred to our hospital for the evaluation of an abnormal finding on chest X‐ray. Chest computed tomography (CT) revealed a 31 × 11 mm mass shadow in the right upper lobe and enlarged mediastinal lymph nodes. Positron emission tomography/computed tomography (PET/CT) showed active uptake of 2‐deoxy‐2‐[^18^F]fluoro‐D‐glucose (FDG) into the primary tumor and mediastinal lymph nodes. A transbronchial biopsy of the primary site was performed, and the tumor was diagnosed as SCLC, exhibiting small and round cells with scant cytoplasm and finely granular nuclear chromatin, with positive immunostaining for synaptophysin, chromogranin, and CD56. An enlarged superior mediastinal lymph node was also diagnosed as a metastasis of SCLC by endobronchial ultrasound transbronchial needle aspiration. No brain metastasis was identified on magnetic resonance imaging (MRI), and the patient was finally diagnosed with LD‐SCLC (c‐T2aN2M0, stage IIIA). Biopsy specimens of the primary tumor were positive for ASCL1 (ASCL1‐high) but negative for NEUROD1 (NEUROD1‐low), as shown in Figure 1a.

**FIGURE 2:**
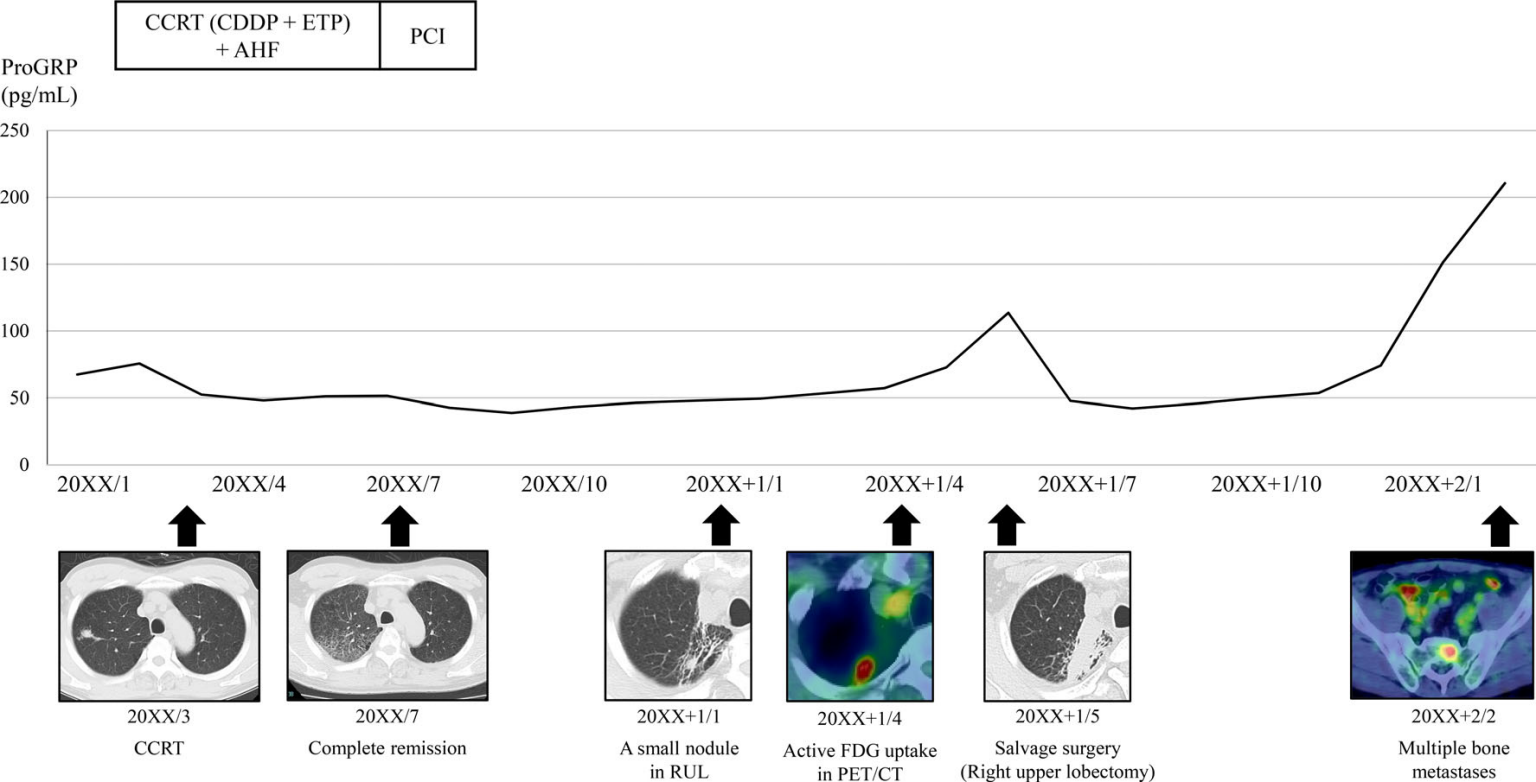
Clinical course. Concurrent chemoradiotherapy (CCRT) was initially performed for limited disease small cell lung cancer (LD‐SCLC) in the right upper lobe, resulting in complete remission. Approximately 6 months later, a small recurrent nodule was detected in the same lobe with active 2‐deoxy‐2‐[^18^F]fluoro‐D‐glucose (FDG) uptake on positron emission tomography/computed tomography (PET/CT). Salvage surgery was performed to control local recurrence. Although the patient was subsequently followed up without postoperative treatment, progastrin‐releasing peptide (ProGRP) levels were again elevated. Multiple bone metastases were detected 9 months after the surgery.

The patient received CCRT comprising four cycles of cisplatin (80 mg/m^2^, day 1) and etoposide (100 mg/m^2^, days 1–3) in combination with accelerated hyperfractionated radiotherapy (45 Gray/30 times). Four months after CCRT, a chest CT scan showed that the tumor and swollen mediastinal lymph nodes had markedly shrunk, and complete remission (CR) was achieved. After additional prophylactic cranial irradiation, the patient developed mild radiation pneumonitis but survived without cancer recurrence. Six months after the completion of treatment, a follow‐up chest CT revealed a small nodule within the lesion of radiation pneumonitis in the right upper lobe, which gradually enlarged. Although transbronchial biopsy of the nodule revealed no malignant findings, it indicated a local recurrence of SCLC because PET/CT showed FDG uptake in the nodule and serum levels of progastrin‐releasing peptide (ProGRP) were elevated (113.6 pg/mL). After a thorough examination, the lesion was determined to be confined to the right upper lobe, and the patient underwent salvage surgery with right upper lobectomy and mediastinal lymph node dissection. Histological examination revealed that the tumor was a recurrence of SCLC with no lymph node metastasis (p‐T1cN0M0, stageIA3), and immunohistochemical staining showed consistently positive results for chromogranin A, synaptophysin, and CD56. The surgical specimens of the recurrent tumor were positive for both ASCL1 and NEUROD1 (ASCL1‐high and NEUROD1‐high), which suggested that the alterations in the expression of these critical lineage transcription factors reflected a transition from neuroendocrine‐high to neuroendocrine‐low SCLC (Figure 1b). The patient was subsequently followed‐up without postoperative treatment, but unfortunately, the serum levels of ProGRP were markedly elevated again, and multiple bone metastases were detected by PET/CT 9 months after the surgery (Figure 2).

## DISCUSSION

Recent studies have classified SCLC into four groups based on its molecular biological characteristics and the RNA or protein expression of lineage transcription factors ASCL1, NEUROD1, YAP1, and POU2F3, with more than four‐fifths of SCLC tumors belonging to the neuroendocrine‐high SCLC‐ASCL1 or SCLC‐NEUROD1 subtypes.^4^, ^5^ ASCL1 is expressed in most SCLC tumors and its expression is tightly linked to neuroendocrine differentiation, while NEUROD1 plays an important role in the development and maturation of various neuronal systems as well as in the formation of neuroendocrine morphology. Basically, these two transcription factors have been found to have predominantly exclusive expression, with only a low degree of coexpression.^4^, ^7^ However, the dynamic transition of its neuroendocrine differentiation, such as from SCLC‐ASCL1 to SCLC‐NEUROD1 due to changes in Myc proto‐oncogene protein (MYC), has recently been reported.^8^, ^9^ These transitions have been primarily demonstrated in vitro, and there are several pieces of evidence for the transitions in molecular subtypes during SCLC evolution in the clinical setting because SCLC patients rarely undergo surgical resection or rebiopsy after disease recurrence.

In our case, two pathological tissues comprising specimens taken at initial diagnosis and from salvage surgery were obtained during the clinical course. The biopsy specimen was originally ASCL1‐high and NEUROD1‐low; however, the surgical specimen became ASCL1‐high and NEUROD1‐high. The patient had a bone metastatic recurrence in the short term after salvage surgery; therefore, the locally recurrent lesion that had transformed from NEUROD1‐low to NEUROD1‐high was likely to have become more malignant than at initial presentation. Several studies have reported that NEUROD1‐expressing SCLC is highly malignant and has a poor prognosis. Zhang et al. identified that NEUROD1‐high SCLC cells show reduced epithelial features and lack EPCAM expression, exhibiting a higher metastatic capability than ASCL1‐high SCLC cells.^10^ Ikematsu et al. analyzed the effects of overexpression or depletion of NEUROD1 on cell migration in SCLC cell lines. They revealed that NEUROD1 expression may occur during the transition from classical to variant forms of SCLC, and that the transition accounts for the process of tumor growth and progression; therefore, NEUROD1 promotes the migration of SCLC cells and might drive SCLC metastasis.^11^

Although the detailed mechanism for the transition of SCLC molecular subtypes is unknown, it is speculated that epithelial–mesenchymal transition (EMT) with the activation of the Notch, Hippo, and TGFβ pathways and MYC oncogene is associated with this transition because EMT would be induced by exposure to chemotherapy or chemoradiotherapy.^12^, ^13^ In our case, there was a possibility that the alterations in molecular subtypes from NEUROD1‐low to NEUROD1‐high may have promoted the relapse after CCRT and the distant metastasis after salvage surgery. In other words, it would be assumed that the microtumor cells remaining after CCRT grew as local recurrence while changing the intratumoral heterogeneity. Further accumulation of similar cases is needed to clarify this point.

There are some limitations to this case. First, it was likely that the entire lesion of the tumor had not been evaluated at the time of initial diagnosis because the molecular subtype evaluation was performed on tiny biopsy specimens. Second, only the molecular subtype of SCLC was evaluated, not MYC or other molecules that may be upregulated in conjunction with the subtype transition.

In summary, we performed salvage surgery for the local recurrence of SCLC after a CR was obtained by CCRT, and histological examination confirmed the alterations in molecular subtypes. The shift to NEUROD1‐high expression in tumor cells surviving CCRT may be related to the poor outcome after combined modality treatment.

## AUTHOR CONTRIBUTIONS

All authors had full access to the data in the study and take responsibility for the integrity of the data and the accuracy of the data analysis. Conceptualization, K.Y., Y.K. and T.H.; Data Curation, K.Y., Y.K., and T.H.; Investigation, K.Y., T.M., Y.O., S.M., Y.K. and T.H.; Formal Analysis, K.Y., T.M., Y.O., S.M., Y.K. and T.H.; Resources, T.M., Y.O., and S.M.; Writing ‐ Original Draft, K.Y. and T.H.; Writing ‐ Review & Editing, K.Y. and T.H.; Visualization, T.H.; Supervision, H.N.; Funding Acquisition, T.H.

## CONFLICT OF INTEREST STATEMENT

The authors have no conflict of interest to declare.
